# Late Onset Bipolar Disorder due to a Lacunar State

**DOI:** 10.1155/2014/780742

**Published:** 2014-03-27

**Authors:** Elena Antelmi, Margherita Fabbri, Lucia Cretella, Maria Guarino, Andrea Stracciari

**Affiliations:** Neurology Unit, S. Orsola-Malpighi University Hospital, Via Albertoni 15, 40138 Bologna, Italy

## Abstract

*Objective.* To describe a patient with a new onset bipolar disorder (BD) type II, secondary to a lacunar state.* Background.* Poststroke BD is rare and mainly associated with lesion in the prefrontal-striatal-thalamic circuit.* Materials and Methods.* A 51-year-old woman came to our attention for a mood disorder of recent onset. At 49, she had suffered acute left-sided limb weakness that improved spontaneously four days later. Arterial hypertension was subsequently diagnosed. After 6 months, she began to suffer from alternating brief periods of expansive and elevated mood with longer periods of depressed mood, with a suicide attempt. We performed extensive laboratory and instrumental investigations, as well as, psychiatric consultation, and a cognitive assessment, which was repeated 9 months later.* Results.* Brain magnetic resonance disclosed leukoaraiosis and a lacunar state of the basal ganglia. Transcranial Doppler showed a patent foramen ovale. A psychiatric consultation led to the diagnosis of BP type II. Neuropsychological evaluation detected deficits in attention/executive functions, verbal fluency, and memory. Nine months later, after specific psychiatric therapy, the psychiatric symptoms were remarkably improved.* Conclusion.* Our case sheds light on the role of the basal ganglia in mood disorders and the importance of ruling out brain injury in late onset BP.

## 1. Introduction

Bipolar disorder (BD) is a mood disorder typically characterized by oscillating manic and depressive states [1]. It is a major source of psychiatric morbidity and mortality affecting approximately 1.5–3% of the population [2]. The etiology of BD remains unknown, though some data support a genetic basis [3]. Secondary forms have seldom been reported in the literature. BD has been reported as a consequence of different medical conditions and after injury to the right dorsolateral prefrontal circuit and anterior cingulate circuit [4].

We describe a 51-year-old woman with a new onset BD type II secondary to a lacunar state of the basal ganglia, analyzing the clinical and neuropsychological features and formulating an anatomopathophysiological hypothesis. The role of neuroimaging in cases of an adult new onset psychiatric disorder is also stressed.

## 2. Case Report

A 51-year-old woman was referred to our hospital for a behaviour and mood disorder that had arisen almost one year before our evaluation together with the complaint of mild memory deficit.

History taking disclosed a sudden onset of left-sided limb weakness at the age of 49 that lasted 4 days, for which patient did not consult a specialist. Subsequent routine medical investigation revealed severe hypertension (of unknown onset) for which she was started on antihypertensives. Six months after the acute episode, she began to present a mood disorder with a change in behaviour. She had at least two episodes of insomnia, distractibility, increased energy, and expansive and elevated mood, circumstantially accompanied by eccentric behaviour (such as bathing while dressed), lasting nearly one week each. These episodes of elevated mood alternated with periods of apathy, loss of interest or decreasing physical energy, and depressive mood. During one of these depressive periods, she even attempted suicide. She denied any personal or family history of psychiatric disorders and her medical history was otherwise unremarkable. History taking from relatives confirmed that the patient had never had previously any psychiatric symptoms or complains.

We performed a neurologic evaluation (NE), laboratory work-up, carotid duplex ultrasound (CDU), transcranial Doppler (TCD), brain magnetic resonance (MR) with gadolinium, psychiatric observation, and a neuropsychological battery of tests comprising an evaluation of attention/executive functions, verbal fluency, and verbal/visual memory (for details of tests adopted, see [5]). Neuropsychological investigations were performed at two different occasions: the first evaluation was done almost one year after the onset of mood disorders, while the second took place nine months after the first evaluation and after specific psychiatric treatment.

**Figure 1:**
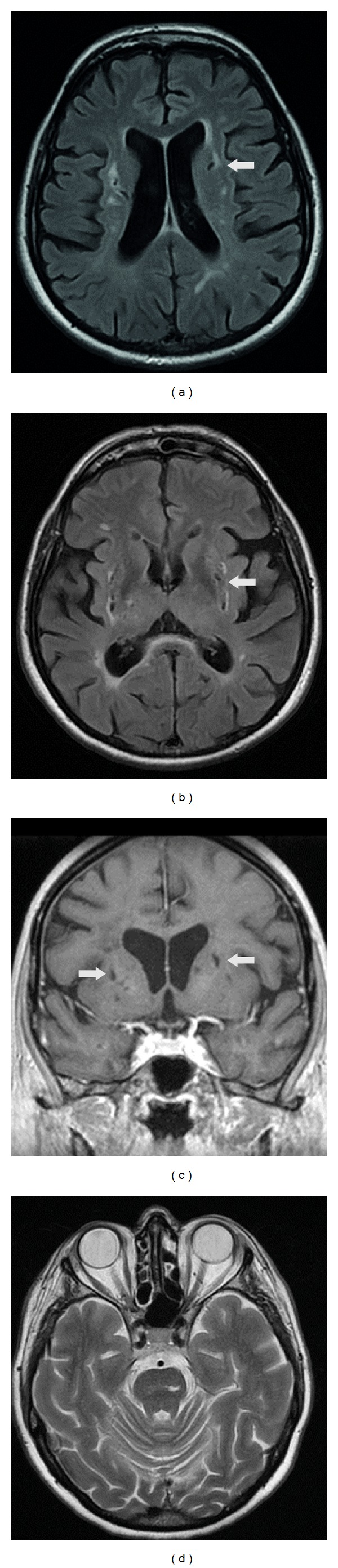
Cerebral MRI: lacunar state, with prominent lesions on the left caudate ((a and c) arrow), bilateral putamen ((b and c) arrow), and left pons (d).

NE disclosed primitive reflexes and a slight bilateral postural hand tremor. Laboratory investigations and CDU were normal. TDC disclosed a patent foramen ovale. Cerebral MR revealed multiple bilateral subcortical lacunar lesions both within the periventricular region and at the nucleus-capsular level resulting in a lacunar state (Figure 1). The psychiatric interview led to diagnosis of type II bipolar disorder, according to the DSM IV TR criteria [1]. The first neuropsychological evaluation disclosed a deficit of attention/execution functions, verbal fluency, and long-/short-term memory (Table 1). By the first assessment, patient was started on valproic acid, quetiapine, and venlafaxine. The dose was progressively augmented to valproic acid 250 mg at 8 a.m. and 500 mg at 8 p.m., quetiapine 300 mg once daily, and venlafaxine 150 mg once daily. The medications offered partial benefit. Therefore, after nine months, venlafaxine and quetiapine were stopped and the patient continued taking only valproic acid 500 mg bd. After nine months, we performed a new clinical and neuropsychological evaluation. Clinically, the patient appeared less depressed and she did not have any further episodes of mania or hypomania. The neuropsychological battery failed to disclose remarkable differences (Table 1). A mild frailty in emotion recognition was detected by Eyes's test [5]. At this time, we also administered Barratt impulsiveness scale 11 (BIS 11) [6] to test for impulsiveness and Yale-Brown obsessive compulsive scale (Y-BOCS) [7] to test for obsessive-compulsive disorder. BIS-11 disclosed a moderate degree of impulsiveness, while Y-BOCS revealed mild obsessive-compulsive traits (Table 1).

## 3. Discussion

Our patient fulfills the DSM-IV criteria for BD type II (having presented at least two hypomanic phases lasting at least one week and an episode of major depression) due to a medical condition with mixed features (293.83) [1]. Indeed, the patient's negative family and personal history for psychiatric disorders, the presence of vascular risk factors, the age of onset and the neuroimaging finding together with the temporal relationship (within two years after the stroke) made a secondary mood disorder more likely, rather than primary mood disorder [1, 4, 8]. In this case, damage to the basal ganglia, together with periventricular white matter changes, which have been described as a predisposing factor, is thought to have led to the mood disorder.

Literature reports have linked different medical conditions to secondary BD, for example, lupus erythematosus, normal pressure hydrocephalus, anoxic encephalopathy or tumour, and stroke lesions. Even though the neural circuits involved in mood regulation are complex, the dorsolateral prefrontal circuit and the anterior cingulate circuit (originating in the prefrontal cortex, projecting to the striatum and thalamus, and then returning to the cortex) seem to be the “core” of mood regulation [8–13]. To support this hypothesis, several studies have shown structural and functional abnormalities within the anterior limbic network, including the basal ganglia, namely, the caudate nucleus. In particular, basal ganglia structures seem to be particularly closely networked with both cortical and subcortical regions involved in mood expression and regulation, including the amygdala and ventral prefrontal cortex, and appear to be critical in affective regulation [9–12]. Several literature reports seem to confirm this role and postlesional mania and BD have been reported after damage to limbic regions of the frontal and temporal lobes and to the basal ganglia, mainly the caudate or pallidum and thalamus, particularly in the right hemisphere [4, 13, 14]. Data confirming the central role of this network in the genesis of BD also emerged from neuroimaging and neurofunctional studies in idiopathic cases. Indeed, increased caudate volume has been widely observed in BD patients compared with matched groups of healthy subjects [15–17]. In addition, functional abnormalities in the basal ganglia, and particularly the caudate, have been widely observed in BD patients using both positron emission tomography and functional magnetic resonance imaging [18]. Similarly, to further support this hypothesis, depression in Parkinson's disease, Huntington's disease, and epilepsy is correlated with reduced metabolic activity in the orbitofrontal cortex and caudate nucleus [19, 20].

Moreover, as already reported [21], the microstructural white matter changes in our patient may have triggered further mood impairment because of a disconnection of cortical and subcortical regions [12].

Another important issue in our case is the cognitive impairment. Cognitive deficits are also recognized as an integral part of the clinical expression of BD. Even if some tasks may improve with euthymia (such as visual and working memory), others tend to persist [22]. Whether the cognitive dysfunctions are related to the mood disturbances and fluctuations or to structural and functional impairment of the same circuits implicated in the mood disorder is still a matter of debate [22]. However, our patient presented cognitive dysfunction related to executive, visual, and verbal memory which persisted in the follow-up evaluation despite mood improvement, confirming the crucial role of the frontostriatal system for adaptive behaviour [12]. Moreover, the cognitive changes in our patient were those typical of subcortical alterations due to a lacunar state, even if other lesions, namely, leukoaraiosis, can also exacerbate cognitive deficits in bipolar disorder.

Finally, several studies have also shown structural and functional involvement of the frontal-subcortical circuit in the pathogenesis of obsessive compulsive disorder [12]. Hence, it is not surprising that the neuropsychological evaluation in our patient disclosed mild impulsiveness and mild obsessive compulsive traits.

In conclusion, damage to the interactive and reciprocal connections between limbic structures and the prefrontal cortex and striatum in our patient may explain the deregulation of emotional control and concomitant dysfunction of executive functions together with mild obsessive-compulsive and impulsiveness traits. Our case therefore supports the role of the basal ganglia in mood disorders and the importance of ruling out brain injury in late onset BD.

## Figures and Tables

**Table 1:** Neuropsychological assessment.

Test	Range	Cutoff	December 15, 2011 score* ES		September 17, 2012 score* ES	
Attention/executive functions						
Attentional matrices (visual search)	0–60	31	**32.75**	**1**	**23.75**	**0**
Stroop test						
Time (s)	∞–0	36.9	**39.25**	**0**	27.25	2
Errors	∞–0	4.2	1.25	3	0	4
Trail making test A: time (s)	∞–0	93	**101**	**0**	**139**	**0**
Trail making test B: time (s)	∞–0	282	**299**	**0**	**352**	**0**
Trail making test B-A: time (s)	∞–0	186	**188**	**0**	**213**	**0**
Picture interpretation test	0–180	180	NA	—	47	3
Eyes's test	0–36	15	NA	—	14.62	—
Reasoning						
Raven's coloured progressive matrices	0–36	18.9	**16.8**	**0**	**15.8**	**0**
Elithorn's maze	0–36	7.75	11.75	2	16	4
Memory						
Verbal						
Rey's 15 words: immediate recall	0–75	28.5	**27.5**	**0**	**12.5**	**0**
Rey's 15 words: delayed recall	0–15	4.7	**3**	**0**	**1**	**0**
Brief story	0–16	4.75	9.65	2	8.75	2
Digit span	0–9	3.75	5.5	4	4.75	3
Nonverbal						
Immediate visual memory	0–22	13.8	**16**	**1**	**16**	**1**
Corsi blocks	0–9	3.75	**3.75**	**1**	4.75	4
Verbal abilities						
Phonological word fluency (F, A, and S)	0–∞	17.35	**21.3**	**1**	**18.3**	**1**
Semantic fluency	0–∞	25	**29**	**1**	**26**	**1**
Spatial cognition						
Freehand copying of drawings	0–12	7.1	9.4	3	8.4	2
Rating scales						
MMSE	0–30	25	*28 *	—	29	—
BIS-11	30–120	—	NA	—	**64**	—
Y-BOCS	0–40	—	NA	—	**10**	—

*Adjusted for age, education, and sex when needed.

ES: equivalent score; NA: not administered.

Defective and borderline scores (ES = 0 and 1, resp.) are in bold type.
